# Survival Outcomes of an Early Intervention Smoking Cessation Treatment After a Cancer Diagnosis

**DOI:** 10.1001/jamaoncol.2024.4890

**Published:** 2024-10-31

**Authors:** Paul M. Cinciripini, George Kypriotakis, Janice A. Blalock, Maher Karam-Hage, Diane M. Beneventi, Jason D. Robinson, Jennifer A. Minnix, Graham W. Warren

**Affiliations:** 1 Department of Behavioral Science, University of Texas MD Anderson Cancer Center, Houston; 2 Department of Radiation Oncology, Medical University of South Carolina, Charleston

## Abstract

**Importance:**

Smoking after a cancer diagnosis increases mortality and risk for a second cancer.

**Objective:**

To determine the association between time of entry into a smoking cessation intervention following a cancer diagnosis and survival outcomes.

**Design, Setting, and Participants:**

Using a prospective cohort study design, patients with cancer who smoked and received cessation treatment were assessed at 3 months, 6 months, and 9 months following tobacco treatment onset. Survival outcomes of tobacco treatment were measured and compared among patients at the MD Anderson Cancer Center Tobacco Research and Treatment Program. Treatment occurred between January 1, 2006, and March 3, 2022. Patients were excluded if they died before the tobacco treatment ended, received their diagnosis more than 6 months after beginning cessation treatment, or lacked staging information. The data analysis took place from September 2023 to May 2024.

**Interventions:**

Cessation treatment consisted of 6 to 8 personalized counseling visits and 10 to 12 weeks of pharmacotherapy. More than 95% of visits were provided via telemedicine.

**Main Outcomes and Measures:**

The primary outcomes were survival as recorded in the MD Anderson Cancer Center tumor registry and 7-day point prevalence abstinence at each follow-up.

**Results:**

The main analytical sample consisted of 4526 currently smoking patients diagnosed with cancer and receiving cessation treatment (2254 [49.8%] female; median [IQR] age, 55 [47-62] years). Survival over 15 years increased for those quitting smoking at 3 months (adjusted hazard ratio [aHR], 0.75 [95% CI, 0.67-0.83]), 6 months (aHR, 0.79 [95% CI, 0.71-0.88]), and 9 months (aHR, 0.85 [95% CI, 0.76-0.95]) of follow-up. The optimal survival outcomes were observed for patients who received tobacco treatment within 6 months of a cancer diagnosis. At the 75th percentile, their survival increased from 2.1 years (95% CI, 1.8-2.4 years) among continuing smokers (nonabstainers) vs 3.9 years (95% CI, 3.2-4.6 years) for patients who quit (abstainers). Similar but less pronounced outcomes were noted when tobacco treatment began within 6 months to 5 years following diagnosis, with survival at the 75th percentile of 4.8 years (95% CI, 4.3-5.3 years) for nonabstainers vs 6.0 years (95% CI, 5.1-7.2 years) for abstainers.

**Conclusions and Relevance:**

The results of this prospective cohort study suggest that evidence-based smoking cessation treatment within 6 months following a cancer diagnosis maximizes survival benefit. This study supports smoking cessation as an important early clinical intervention for patients after being diagnosed with cancer.

## Key Points

**Question:**

Is the timing of patient entry into a smoking cessation program after a cancer diagnosis associated with overall survival?

**Findings:**

In this cohort study of 4526 currently smoking patients diagnosed with cancer, smoking cessation treatment at 3 months, 6 months, or 9 months after initiation was associated with improved survival. Survival outcomes were optimal in patients entering tobacco treatment within 6 months of a cancer diagnosis.

**Meaning:**

Initiation of tobacco treatment within 6 months of a cancer diagnosis resulting in smoking cessation has the largest survival benefit, emphasizing the importance of early postdiagnosis entry into an evidence-based tobacco treatment program.

## Teaser

This cohort study evaluates the survival outcomes of currently smoking patients with cancer who entered into a smoking cessation treatment program based on the time between diagnosis and program entry.

## Introduction

Smoking by patients with cancer and cancer survivors causes adverse cancer treatment outcomes.^1^ The 2014 Surgeon General’s report on tobacco reviewed more than 400 studies between 1992 and 2012, concluding that smoking at or following a cancer diagnosis increases both all-cause and cancer-specific mortality, as well as risk for disease progression and tobacco-related second primary cancers. Across various cancer types, continuing to smoke increased the risk of overall mortality by a median rate of 50% and cancer-related mortality by a median rate of 61%. The adverse effects of smoking on cancer treatment outcomes and costs are observed across cancer disease types.^2^ The 2020 Surgeon General’s report and recent meta-analyses among patients with lung or head and neck cancer support improved survival with quitting smoking among these patients.^3,4,5^ However, a significant limitation noted by these reviews was the lack of clear, consistent smoking data and prospective longitudinal outcomes. Importantly, none of these earlier studies analyzed the association of time between diagnosis and entry into a smoking cessation treatment program with survival.

Although smoking cessation as a part of cancer care is advocated by most large cancer organizations,^6,7^ many oncology clinicians do not regularly assist patients with quitting.^7,8^ The Tobacco Research and Treatment Program (TRTP) at the University of Texas MD Anderson Cancer Center has been providing structured, evidence-based smoking cessation interventions for patients diagnosed with cancer since 2006. Data collected through the TRTP provide a unique opportunity to accurately assess the benefits of quitting smoking after diagnosis.^9^

## Methods

### Study Design and Participants

The purpose of this prospective cohort study was to evaluate the survival outcomes associated with smoking cessation among patients diagnosed with cancer and receiving tobacco treatment within 6 months, from 6 months to 5 years, and more than 5 years after diagnosis. We reported the study results according to the Strengthening the Reporting of Observational Studies in Epidemiology (STROBE) reporting guideline.^10^ The University of Texas MD Anderson Cancer Center institutional review board approved the research as a database protocol, thus granting a waiver of informed consent. Survival analyses were performed among currently smoking patients with cancer who received treatment from the TRTP between January 1, 2006, and March 3, 2022.

### Smoking Cessation Treatment Methods

Established in 2006, the TRTP is funded by the State of Texas Tobacco Settlement Funds, allocated to MD Anderson Cancer Center. TRTP provides smoking cessation treatment at no cost to all patients to remove barriers to care and provide evidence-based treatment among all patients who need assistance with quitting smoking.^9^ The TRTP offers personalized interventions, including cognitive behavioral counseling, motivational interviewing, and pharmacotherapy. Medications provided include nicotine replacement therapy (eg, nicotine patches, gum, and lozenges), bupropion, and varenicline, either as monotherapies or in various combinations.^11^ Patients were enrolled in the TRTP through several means, including a proactive system identifying them as current smokers through the electronic medical record, self-referral, or referral by their clinician. More than 95% of visits in this study were provided via telemedicine. A complete program description with documented outcomes of 35% to 44% abstinence has been previously published.^9,11^

The primary exposure under investigation was self-reported abstinence at 3 months, 6 months, and 9 months following the initial TRTP consultation.^9^ Abstinence information was collected prospectively by TRTP staff (not the treating clinicians), defined using a timeline follow-back method at all visits. All data were promptly entered into the TRTP database.^12^ Abstinence was defined as self-reported no smoking in the 7 days before each assessment (7-day point prevalence). Carbon monoxide verification of abstinence could only be carried out at the in-person visits and showed a 0.9 correlation with self-reporting.^9^ Patients who had missing smoking status at 3 months, 6 months, and 9 months of follow-up were treated as nonabstainers for purposes of the data analysis, using intent-to-treat (ITT), as is standard practice in published literature.^13,14^ We also performed a sensitivity analysis of the ITT method for missing smoking status using multiple imputation methods presented in eAppendix 1 in Supplement 1.^15^

### Methods for Determining Survival

The primary outcome was survival recorded by the MD Anderson Cancer Center tumor registry. Because median survival was not attained among patients, analyses were performed for patients at the 75th percentile (ie, time when 75% of patients survived). To evaluate time-dependent survival outcomes associated with smoking cessation after diagnosis, we conducted the analysis among 3 subgroups based on the time between diagnosis and entry into the TRTP (ie, within 6 months, from 6 months to 5 years, and more than 5 years). Additionally, we evaluated the survival outcomes of the time from diagnosis to entry into the TRTP treating time as continuous (eAppendix 2 in Supplement 1). Survival for all patients, regardless of time of program entry, was also reported.

### Statistical Analysis

The study variables analyzed for this project included age at diagnosis, time of diagnosis, sex, race and ethnicity, cancer diagnosis according to the* International Classification of Diseases for Oncology (ICD-O), third edition*, histologic type, stage at diagnosis, last contact date, vital status, and date of death. Categorical variables were characterized using counts and proportions, while continuous variables were presented with medians and IQRs. Pearson χ^2^ tests were used to analyze categorical variables, and Mann-Whitney U tests were used for continuous variables. All statistical tests were conducted using a 2-sided approach with a 2-tailed *P* value threshold of .05.

Univariate Kaplan-Meier survival analyses were performed for overall survival among all patients and among the subgroups, as defined by the time between cancer diagnosis and entrance into the TRTP. Log-rank statistics were calculated to evaluate differences between abstainers (smokers who quit) and nonabstainers (continuing smokers). All analyses were conducted using Stata, version 18 (StataCorp).^16^

Multivariate Cox proportional hazard regression models were performed and adjusted for age at diagnosis, stage, gender, race and ethnicity, cancer site, and actual time between diagnosis and entering TRTP. Cox models for the total sample, based on time between diagnosis and entry to TRTP, were estimated for abstinence status at 3 months, 6 months, and 9 months. Model fitting was performed using the Schoenfeld residuals.^17^ The Harrell C statistic was calculated to determine the model’s discrimination.^18^ We present the Cox proportional hazards regression model results as adjusted hazard ratios (aHRs) with 95% CIs. Our primary analyses focused on patients with staging information. To assess the consistency of the results when incorporating patients without stage information, we also repeated the analysis on the combined sample of patients with and without staging information, treating those with missing stage data as a fourth category (stage = none; eTables 3 and 4 in Supplement 1).

## Results

### Patient Characteristics

**Figure 1.**
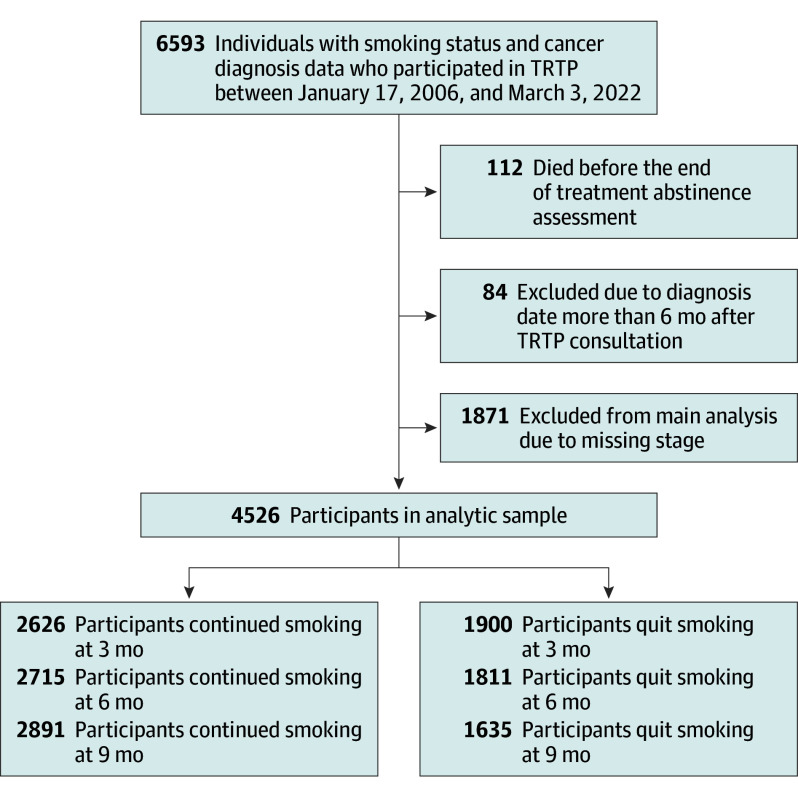
Study Flow Diagram Currently smoking patients with cancer in the Tobacco Research and Treatment Program (TRTP) at the University of Texas MD Anderson Cancer Center were enrolled in the study. After the exclusions noted in the flow diagram, 4526 individuals remained in the analytic sample.

Among 6593 currently smoking patients who underwent tobacco treatment in the TRTP, the primary analytic sample consisted of 4526 patients (Figure 1). Individuals were excluded if they died before the end of cessation treatment (3-month follow-up; 112 patients) as no data were available for 3 months, 6 months, and 9 months of follow-up. Those diagnosed more than 6 months after joining the TRTP (84 patients) were also excluded. Patients without staging information were not included in the main analysis (n = 1871); however, a survival outcome analysis including both patients with and without staging information is provided in eTables 3 and 4 in Supplement 1.

Among the patients who were analyzed, 2254 (49.8%) were female, and the median (IQR) age was 55 (47-62) years. Basic demographics for the sample with staging information are provided in the Table, including tobacco use, dependence (Fagerström Test for Cigarette Dependence), disease site, and stage.^19,20^ *ICD-O*–specific cancer sites, consisting of the groups shown in the Table, are provided in eTable 1 in Supplement 1. Most patients were diagnosed with breast cancer (n = 790; 17.5%), lung cancer (n = 782; 17.3%), head and neck cancer (n = 587; 13.0%), or hematologic cancer (n = 375; 8.3%). The median (IQR) follow-up time was 7.9 (3.3-11.8) years. As shown in eTable 2 in Supplement 1, overall ITT abstinence rates across this sample were 42% (1900/4526) at 3 months, 40% (1811/4526) at 6 months, and 36% (1635/4526) at 9 months. Analyses of respondent-only data indicated abstinence rates of 47% (1900/4009) at 3 months, 50% (1811/3613) at 6 months, and 50% (1635/3255) at 9 months (eTable 2 in Supplement 1).

**Table.** Characteristics of Patient Sample

Characteristic	Patients, No. (%) (N = 4526)
Sex	
Female	2254 (49.8)
Male	2272 (50.2)
Race and ethnicity	
Black	509 (11.2)
White	3537 (78.1)
Other^a^	480 (10.6)
Disease site	
Breast	790 (17.5)
Lung	782 (17.3)
Head and neck	587 (13.0)
Hematologic	375 (8.3)
Genitourinary	363 (8.0)
Skin	301 (6.7)
Prostate	285 (6.3)
Gynecologic	203 (4.5)
Rectum	151 (3.3)
Esophagus	123 (2.7)
Endocrine	105 (2.3)
Colon	103 (2.3)
Musculoskeletal	86 (1.9)
Pancreas	56 (1.2)
Liver	43 (1.0)
Gastrointestinal	38 (0.8)
CNS	36 (0.8)
Anal	33 (0.7)
Abdomen	26 (0.6)
Eye/ear	20 (0.4)
Unknown	13 (0.3)
Biliary	7 (0.2)
Stage	
I/II	2131 (47.1)
III	1064 (23.5)
IV	1331 (29.4)
Age at diagnosis, median (IQR), y	55 (47-62)
Pack-years, median (IQR)	23 (0-40)
Cigarettes per day, median (IQR)	15 (9-20)
FTCD, median (IQR)^19^	4 (3-6)

Abbreviations: CNS, Central Nervous System; FTCD, Fagerström Test for Cigarette Dependence.

^a^ Other race includes American Indian, Alaska Native, Asian, Filipino, Hawaiian, and unknown.

Baseline characteristics and abstinence rates of 6397 patients in the combined sample of those with and without staging information are shown in eTables 3 and 4 in Supplement 1. The demographics and abstinence rates for the sample of patients with staging data and the combined sample of patients with and without staging data were similar. In addition, as outlined in eAppendix 3 in Supplement 1, the study sample was representative of the US population on several socioeconomic factors related to social determinants of health, and other studies of smoking cessation among patients with cancer.^21,22,23^

### Association of Abstinence With Survival

**Figure 2.**
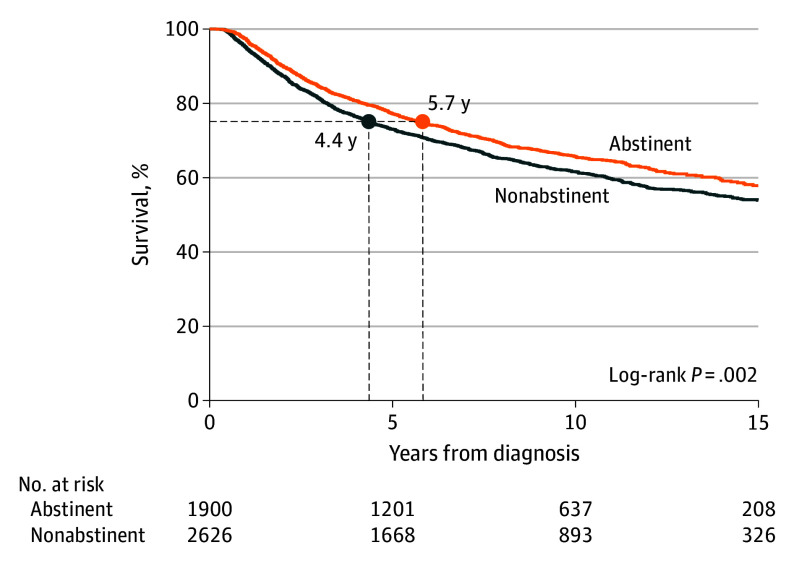
Overall Survival Outcomes Associated With Smoking Cessation at 3 Months The survival outcomes at 3 months were shown without respect to time from diagnosis to entry into the tobacco cessation program among patients with stage information (N = 4526).

Patients who had missing smoking status at 3 months (517 [11%]), 6 months (913 [20%]), and 9 months (1271 [28%]) of follow-up were treated as nonabstainers. As shown in Figure 2, abstinence vs nonabstinence at 3 months among the primary cohort (N = 4526) was associated with improved survival at 5 years and 10 years (65% vs 61% and 77% vs 73%; *P* = .002). The minimum percentile of survival for the overall cohort was the 56th percentile; thus, the median survival time could not be estimated. Estimating survival at the 75th percentile demonstrated time to death was 4.4 years (95% CI, 3.9-4.9 years) for nonabstainers vs 5.7 years (95% CI, 5.1-6.5 years) for abstainers at 3 months (Figure 2). Survival over 15 years increased for those quitting smoking at 3 months (aHR, 0.75 [95% CI, 0.67-0.83]), 6 months (aHR, 0.79 [95% CI, 0.71-0.88]), and 9 months (aHR, 0.85 [95% CI, 0.76-0.95]) of follow-up.

### Time Between Cancer Diagnosis and TRTP Entry

**Figure 3.**
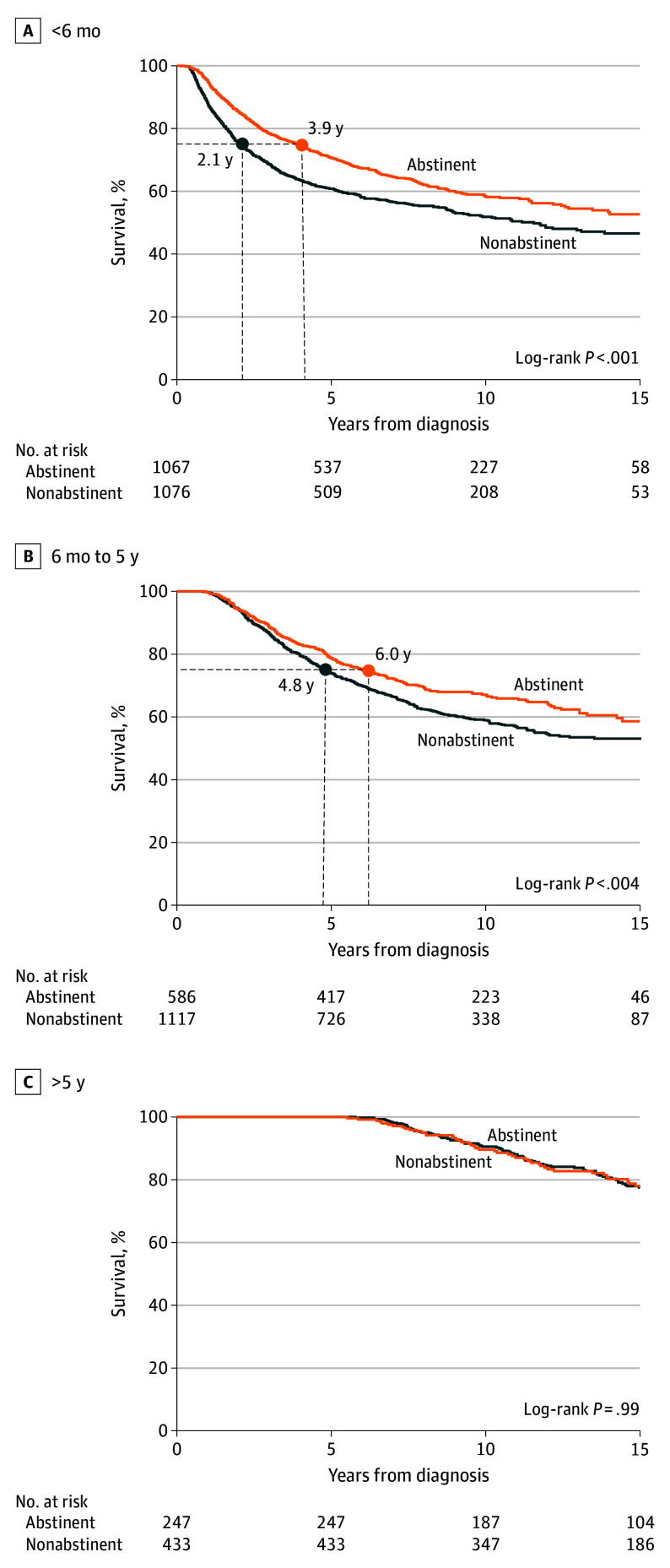
Survival Outcomes Based on Time Between Cancer Diagnosis and Tobacco Cessation Program Entry at 3 Months The survival outcomes at 3 months were shown based on the time between cancer diagnosis and entry into the tobacco cessation program among patients with stage information (N = 4526).

The association of time between diagnosis and entry into the TRTP with survival outcomes for the primary cohort is shown in Figure 3. For patients entering the TRTP within 6 months of diagnosis, abstinence vs nonabstinence at 3 months was associated with improved survival at 5 years and 10 years (61% vs 71% and 52% vs 58%, respectively; *P* < .001), with continued benefits observed through 15 years. A similar association was observed among patients who entered the TRTP between 6 months and 5 years from diagnosis (10-year survival from TRTP entry for abstainers vs nonabstainers: 59% vs 67%; *P* = .004). No significant association with survival was noted among patients entering the TRTP more than 5 years after diagnosis (10-year survival from TRTP entry for abstainers vs nonabstainers: 91% vs 90%; *P* = .99). Similarly, the association between continuous time from diagnosis to TRTP entry and survival is provided in eAppendix 2, the eFigure, and eTable 5 in Supplement 1. These findings are consistent with those in the main analysis and illustrate the diminishing impact of abstinence after each passing month.

Among patients with cancer entering the TRTP within 6 months of diagnosis, survival at the 75th percentile increased from 2.1 years (95% CI, 1.8-2.4 years) among nonabstainers vs 3.9 years (95% CI, 3.2-4.6 years) for abstainers (Figure 3). Among patients entering TRTP between 6 months and 5 years from diagnosis, survival at the 75th percentile was 4.8 years (95% CI, 4.3-5.3 years) for nonabstainers vs 6.0 years (95% CI, 5.1-7.2 years) for abstainers.

### Abstinence at 3 Months, 6 Months, and 9 Months After TRTP Entry

**Figure 4.**
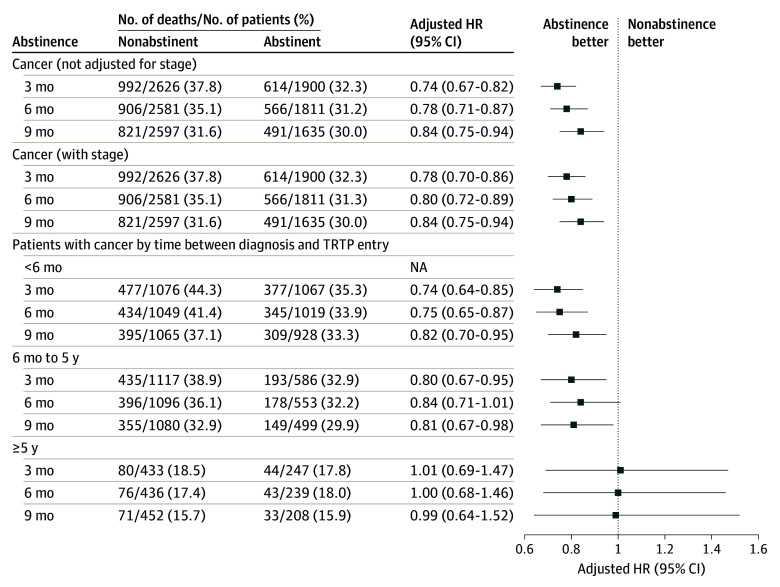
Survival Multivariate Analyses According to Cancer Diagnosis and Time Between Diagnosis and Tobacco Cessation Program Entry Survival outcomes were analyzed according to cancer diagnosis and time between diagnosis and entry into the Tobacco Research and Treatment Program (TRTP) at the University of Texas MD Anderson Cancer Center for patients with stage information (N = 4526). HR indicates hazard ratio.

Multivariate Cox regression analyses for the primary cohort without adjusting for stage demonstrated that abstinence at 3 months, 6 months, and 9 months was associated with reduced mortality by 26%, 22%, and 16%, respectively (Figure 4). When adjusting for the potential effects of staging, abstinence at 3 months, 6 months, and 9 months was associated with reduced mortality by 22%, 20%, and 16%.

The association of time between diagnosis and TRTP entry with survival demonstrated abstinence at 3 months significantly reduced mortality by 26% for patients with TRTP entry within 6 months of diagnosis vs 20% between 6 months and 5 years of diagnosis (Figure 4). Abstinence at 6 months reduced mortality by 25% and 16% for entry within 6 months of diagnosis vs between 6 months and 5 years of diagnosis, respectively, and abstinence at 9 months reduced mortality by 18% to 19%, respectively. No significant association was noted for patients entering the TRTP more than 5 years after diagnosis. The results of the multiple imputation analysis are consistent with the ITT results presented herein (eTable 6 in Supplement 1).

To evaluate whether survival benefits could be observed independent of stage, analyses were performed among 6397 patients, including those without staging information (eAppendix 1 and eTable 7 in Supplement 1). Survival benefits associated with abstinence at all time points, with and without adjustment for stage, were highly similar for the larger cohort that included those without staging information, as shown in eTable 7 in Supplement 1.

## Discussion

This cohort study provided clear evidence that quitting smoking at or following a cancer diagnosis improves survival across various types of cancer. The largest benefit was noted among patients who entered the TRTP within 6 months of diagnosis, with 1.8 years of added life (75th survival percentile) among patients abstinent at 3 months. Data also demonstrated that smoking cessation, regardless of time of entry, was associated with improved survival: abstinence at 3 months, 6 months, and 9 months after tobacco treatment onset reduced mortality across all cancer types by 26%, 22%, and 16%, respectively.

To our knowledge, these results provide the strongest evidence to date of the importance of receiving evidence-based tobacco treatment as early as possible following a cancer diagnosis. Moreover, this prospective study overcomes the limitations of other survival studies that rely on a retrospective assessment of abstinence status and lack detailed smoking data (ie, 7-day point prevalence abstinence across multiple time points).^3,4,5^ This study involved a prospective assessment of smoking status among patients with cancer, with multiple real-time standardized assessments of abstinence that more precisely capture the effects of smoking cessation on survival and allow for the quantification of survival benefits arising from early tobacco treatment. Using the 75th percentile for survival, quitting smoking for patients receiving evidence-based tobacco treatment within 6 months of diagnosis added 1.8 years of life compared to patients who continued to smoke. Moreover, the benefit of smoking cessation was observed among patients who quit smoking at 3 months, 6 months, and 9 months, with the greatest benefit for those quitting at 3 months. The positive impact of cessation was observed across a broad cancer cohort even after adjustment for age, stage, gender, and diagnosis. A lesser impact was noted among patients receiving tobacco treatment more than 6 months from diagnosis. These findings were replicated using the sample that included only patients with staging data, as well as a sample including both patients with and without staging data. Collectively, the results show a clear, consistent, and robust improvement in survival by an earlier entry into tobacco treatment after a cancer diagnosis.

These results parallel recent observations from disease-specific analyses demonstrating survival benefits associated with smoking cessation, including a prospective cohort study of patients with lung cancer in the UK showing that quitting smoking reduced mortality by between 25% and 33% and another in Russia showing a 51% reduction in mortality.^24,25^ However, although these studies involved prospective data collection of smoking behavior, no clear smoking cessation intervention was described, and the time between diagnosis and tobacco intervention was not examined. Similarly, most recent meta-analyses documenting the benefits of smoking cessation across lung, head and neck, gastrointestinal, and bladder cancers do not provide clearly defined assessments of smoking status, prospective data collection, or tobacco treatment information, as done in this study.^4,5,26,27^ Moreover, several studies evaluating the benefits of cessation after diagnosis are limited by excluding patients who died within the first several years after diagnosis, thereby eliminating the ability to identify patients whose smoking caused early death.^28,29^ The design and execution of the current study overcomes these prior methodological limitations, confirming the long-term survival benefit of quitting smoking after a cancer diagnosis and extending previous findings that early intervention optimizes survival. Thus, the larger magnitude of survival benefit among patients who received treatment within 6 months of diagnosis suggests that cessation can attenuate mortality risks caused by smoking even within the first few years after diagnosis.

An important clinical implication from this study is that providing a structured smoking cessation program at the time of a cancer diagnosis that is integrated with cancer care can have a demonstrable positive association with life expectancy for patients. Large cancer organizations have been called on to make meaningful cancer advances that will substantially benefit patients, and investing in smoking cessation treatment programs represents an approach that should be a standard component of cancer treatment.^30^ Regulatory agencies that provide expedited approval of new cancer therapies emphasize treatments leading to measurable clinical benefit.^31^ Ensuring that all patients with cancer who smoke have access to smoking cessation treatment can optimize the effectiveness of cancer treatments and contribute to a substantial benefit in survival, in addition to improved non–cancer-related health outcomes.^1,2,3,7^ The results of this study suggest that early entry into tobacco treatment is associated with the best survival outcomes and justifies the need to prioritize tobacco cessation as a core element of first-line cancer care.

### Limitations

Although, to our knowledge, these data represent the most definitive analysis of the relationship between smoking cessation and survival among patients with cancer to date, limitations are present with this study. As with any tumor registry, noncancer information and treatments were not available for the analysis of potential interactions with non–cancer-related health conditions. However, demonstrating survival benefits across multiple cancer types, after adjustment for several important covariates, implies that smoking cessation after diagnosis is a broad outcome modifier for patients with cancer overall.

Another limitation is the possibility that participants in an institutionally sponsored TRTP available to all patients who smoke are not fully representative of patients who smoke with a cancer diagnosis, traditionally including smoking-related cancers, such as lung and head and neck cancers. However, approximately 50% of incident cancers consist of lung, prostate, breast, or colorectal cancers in the general population, which is similar to the 47% of cancers across these major disease sites observed in this study cohort.^32^ Also, the patients in this study all elected to receive tobacco treatment, representing approximately 50% of individuals who were contacted, which may be another limitation.

Nonetheless, abstinence is the defining factor contributing to survival, regardless of how it is achieved, whether in a structured cessation program or on one’s own. Having such a program available for patients enhances the chances of quitting just as participation in evidence-based smoking cessation treatment does for all people who smoke, which can lead to a clinically meaningful survival benefit.^33^ Although abstinence in this study was based on self-reported information, the data were highly correlated with biochemical verification when possible. The relationship between quitting smoking, entry time into the cessation program, and survival was robust and observed over multiple analytic strategies.

## Conclusions

The results of this prospective cohort study provide support for the survival benefit of quitting smoking after a cancer diagnosis and entry into a tobacco cessation program, with the largest survival benefits observed for patients who enter evidence-based cessation treatment within 6 months of the cancer diagnosis. Advances that substantially improve delivery and effectiveness of evidence-based smoking cessation treatment at the time of diagnosis are expected to provide significant survival benefit to patients and value to oncology clinicians.

## Notes

Supplement 1. eAppendix 1. Sensitivity Analysis of ITT Using Multiple Imputation eAppendix 2. Evaluation of Whether Time from Diagnosis to TRTP relates to the effect of abstinence on survival eAppendix 3. Socioeconomic Characteristics of the Sample eTable 1. ICD-O Cancer Site Classification Comprising Cancer Diagnosis Groups Used in the Main Outcome Analysis (n=4526) eTable 2. 3-, 6- and 9-month abstinence rates for all patients with staging and by time of entry into the TRTP for the patients with stage (n=4526) eTable 3. Patient characteristics for both staged and non-staged patients eTable 4. 3-, 6- and 9-month abstinence rates for all patients and time of entry into the TRTP for all (staged and non-staged patients, n= 6,397) eTable 5. Difference in Abstinence Hazard Rates by Time for Cancer Dx to TRTP eTable 6. Multivariate Analyses for Survival According to Cancer Diagnosis and Time between Diagnosis and Entry into the TRTP for the Primary Cohort of Patients with Stage (N=4,426) Using Multiple Imputation eTable 7. Multivariate analyses for survival according to cancer diagnosis and time between diagnosis and entry into the TRTP for the pooled sample (staged and non-staged patients (n=6,397)) eFigure. Difference in abstinence hazard rates by time from diagnosis to TRTP by cohort eReferences

Supplement 2. Data Sharing Statement
