# Supplementary material for: Survival Outcomes of an Early Intervention Smoking Cessation Treatment After a Cancer Diagnosis
Source: JAMA Oncol. 2024 Oct 31;10(12):1689–96. doi: 10.1001/jamaoncol.2024.4890 (PMC11528342; doi:10.1001/jamaoncol.2024.4890)
Supplement: Supplement 1. — eAppendix 1. Sensitivity Analysis of ITT Using Multiple Imputation eAppendix 2. Evaluation of Whether Time from Diagnosis to TRTP relates to the effect of abstinence on survival eAppendix 3. Socioeconomic Characteristics of the Sample eTable 1. ICD-O Cancer Site Classification Comprising Cancer Diagnosis Groups Used in the Main Outcome Analysis (n=4526) eTable 2. 3-, 6- and 9-month abstinence rates for all patients with staging and by time of entry into the TRTP for the patients with stage (n=4526) eTable 3. Patient characteristics for both staged and non-staged patients eTable 4. 3-, 6- and 9-month abstinence rates for all patients and time of entry into the TRTP for all (staged and non-staged patients, n= 6,397) eTable 5. Difference in Abstinence Hazard Rates by Time for Cancer Dx to TRTP eTable 6. Multivariate Analyses for Survival According to Cancer Diagnosis and Time between Diagnosis and Entry into the TRTP for the Primary Cohort of Patients with Stage (N=4,426) Using Multiple Imputation eTable 7. Multivariate analyses for survival according to cancer diagnosis and time between diagnosis and entry into the TRTP for the pooled sample (staged and non-staged patients (n=6,397)) eFigure. Difference in abstinence hazard rates by time from diagnosis to TRTP by cohort eReferences [file jamaoncol-e244890-s001.pdf]

Supplemental Online Content

Cinciripini PM, Kypriotakis G, Blalock JA, et al. Early Intervention Smoking Cessation Treatment After a Cancer Diagnosis. *JAMA Oncol.* Published online October 31, 2024. doi:10.1001/jamaoncol.2024.4890

eAppendix 1. Sensitivity Analysis of ITT Using Multiple Imputation

eAppendix 2. Evaluation of Whether Time from Diagnosis to TRTP relates to the effect of abstinence on survival

eAppendix 3. Socioeconomic Characteristics of the Sample

eTable 1. ICD-O Cancer Site Classification Comprising Cancer Diagnosis Groups Used in the Main Outcome Analysis (n=4526)

eTable 2. 3-, 6- and 9-month abstinence rates for all patients with staging and by time of entry into the TRTP for the patients with stage (n=4526)

eTable 3. Patient characteristics for both staged and non-staged patients

eTable 4. 3-, 6- and 9-month abstinence rates for all patients and time of entry into the TRTP for all (staged and non-staged patients, n= 6,397)

eTable 5. Difference in Abstinence Hazard Rates by Time for Cancer Dx to TRTP

eTable 6. Multivariate Analyses for Survival According to Cancer Diagnosis and Time between Diagnosis and Entry into the TRTP for the Primary Cohort of Patients with Stage (N=4,426) Using Multiple Imputation

eTable 7. Multivariate analyses for survival according to cancer diagnosis and time between diagnosis and entry into the TRTP for the pooled sample (staged and non-staged patients (n=6,397))

eFigure. Difference in abstinence hazard rates by time from diagnosis to TRTP by cohort

eReferences

This supplemental material has been provided by the authors to give readers additional information about their work.

© 2024 Cinciripini PM et al. *JAMA Oncology.*

Appendix

eAppendix 1: Sensitivity Analysis of ITT Using Multiple Imputation

To implement a sensitivity analysis of the abstinence ITT (Intention-To-Treat) imputation approach, we reanalyzed the models presented in Table 2 of the main manuscript using multiple imputation. We have successfully implemented this method in earlier publication using TRTP data[12]. This method is recommended as a primary alternative to ITT[3] . We created 40 imputed datasets using Stata's "mi impute chained" command, which employs a sequential regression multivariate imputation approach to impute one variable at a time, conditioning on all other variables. This approach uses a Gibbs-like algorithm to obtain imputed values by simulating from the posterior predictive distribution. All imputations were performed separately for each cancer site category and were subsequently combined. This nested multiple imputation method has been shown to produce reliable estimates and standard errors with nonignorable missing smoking data[4]. Results from the multiple imputation datasets were analyzed and combined using Stata's "mi estimate" command. Our imputation accounted for cancer group membership by multiply imputing abstinence nested within cancer site categories. We present in **eTable 6** the results of the Cox regression models using multiple imputation[5]. This approach provides a robust sensitivity analysis framework to validate the findings of our main ITT analysis.

© 2024 Cinciripini PM et al. JAMA Oncology.

eAppendix 2: Evaluation of Whether Time from Diagnosis to TRTP relates to the effect of abstinence on survival.

The crucial question in our setting is whether the proximity of receiving smoking cessation treatment to cancer diagnosis modifies the relationship of abstinence on survival. In addition to estimating the effect of abstinence on survival by the three groups (< 6 months, 6 – 5 years, and 5 + years), we also calculated the effect of abstinence as a function of time from diagnosis to TRTP as a continuous modifier. e**Table 7** presents the difference between the hazard rate of abstinent and non-abstinent individuals by month from diagnosis to TRTP, along with the associated p-value and confidence intervals. The longer the distance from diagnosis, the smaller the effect of abstinence on survival. The effect of abstinence, despite gradually reducing with every extra month, remains significant up to the 51st month (4.25 years) after diagnosis. e**Figure 1** presents this relationship and also superimposes the grouping of time from diagnosis as defined in the manuscript. The shaded areas represent the 95% confidence intervals around the estimated effect, and the vertical dashed lines indicate the boundaries of the predefined time groups. The categories '< 6 months', '6 months - 5 years', and '5 + years' are clearly marked to illustrate the decreasing effect of abstinence over time.

© 2024 Cinciripini PM et al. JAMA Oncology.

eAppendix-3 Socioeconomic Characteristics of the Sample:

To further evaluate the socioeconomic characteristics of our sample, we collected both insurance information at the patient level, provided by the MD Anderson's institutional database, and social determinants of health (SDOH) data at the census tract level provided by the CDC[6]. We geocoded our participants' residential addresses to assign census tracts and compare SDOH indicators between our sample average and the national average. In terms of insurance, our sample has substantial representation of private, Medicare, and self-pay participants (see below). For SDOH, we utilized six census tract-level indicators: income, unemployment, Gini index, poverty rate, percentage of Black Non-Hispanic individuals, and percentage of Hispanic individuals. Our sample's census tract indicators for income, unemployment, Gini index, and poverty closely mirror national rates. However, the percentages of Black Non-Hispanic and Hispanic individuals are overrepresented in our sample. This discrepancy is attributed to the geographic focus of our sampling, which primarily encompassed the Greater Houston Area, known for its high diversity.

- **Insurance:**
  - Private: 1,794 (28.04%)
  - Self-pay: 2,047 (32.00%)
  - Medicaid: 166 (2.59%)
  - Medicare: 2,338 (36.55%)
  - Government Other: 50 (0.78%)
  - Missing: 2 (0.03%)

Census Tract-Level Data: To provide additional context, we analyzed neighborhood-level data on income, Black Non-Hispanic, unemployment, Gini Index, and poverty levels. The neighborhoods from which our study population is drawn have the following characteristics:

- **Income:**
  - Median household income is $63,750 for our sample, which is similar to the median income at the national level ($62,557), indicating comparable economic characteristics.
- **Unemployment:**
  - Unemployment rate in the census tracts that our participants reside is 4.85%, similar to the national averages of 4.60%."
- **Gini Index:**
  - The median Gini Index in our study’s census tracts is 4.10%, similar to the national average of 4.10%."
- **Poverty:**
  - The median poverty rates in our study census tracts is 10.03%, very close to the national average of 10.23%."
- **Black Non-Hispanic:**
  - Black-Non-Hispanics constitute 7.07% in our study’s census tracts, overrepresenting the general population's rate of 3.89%.
- **Hispanic:**
  - Hispanic constitute 24.29% in the study’s census tracts, overrepresenting the general population's rate of 7.90%.

© 2024 Cinciripini PM et al. JAMA Oncology.

Supplementary Tables

eTable 1. ICD-O Cancer Site Classification Comprising Cancer Diagnosis Groups Used in the Main Outcome Analysis (n=4526)

	Abdomen	Anal	Biliary	Breast	CNS	Colon	Endocrine	Esophagus	Eye/ear	GI	GU	Gyn	H/N	Heme	Liver	Lung	MSK	Pancreas	Prostate	Rectum	Skin	Unknown	All
	(N=26)	(N=33)	(N=7)	(N=790)	(N=36)	(N=103)	(N=105)	(N=123)	(N=20)	(N=38)	(N=363)	(N=203)	(N=587)	(N=375)	(N=43)	(N=782)	(N=86)	(N=56)	(N=285)	(N=151)	(N=301)	(N=13)	(N=4,526)
TR ICD-O Site Description	n (%)	n (%)	n (%)	n (%)	n (%)	n (%)	n (%)	n (%)	n (%)	n (%)	n (%)	n (%)	n (%)	n (%)	n (%)	n (%)	n (%)	n (%)	n (%)	n (%)	n (%)	n (%)	n (%)
ADRENAL GLAND, NOS							5 (4.8)																5 (0.1)
AMPULLA OF VATER																		4 (7.1)					4 (0.1)
ANAL CANAL		24 (72.7)																					24 (0.5)
ANTERIOR WALL OF BLADDER											3 (0.8)												3 (0.1)
ANTERIOR 2/3 OF TONGUE, NOS													38 (6.5)										38 (0.8)
ANTERIOR FLOOR OF MOUTH													22 (3.7)										22 (0.5)
ANTERIOR MEDIASTINUM																7 (0.9)							7 (0.2)
ANTERIOR SURFACE OF EPIGLOTTIS													3 (0.5)										3 (0.1)
ANUS AND ANAL CANAL, OVERLAP.LESION		2 (6.1)																		4 (2.6)			6 (0.1)
ANUS, NOS (EXCL.SKIN)		6 (18.2)																					6 (0.1)
APPENDIX						13 (12.6)																	13 (0.3)
ASCENDING COLON						9 (8.7)																	9 (0.2)
AXILLARY TAIL OF BREAST				4 (0.5)																			4 (0.1)
BASE OF TONGUE, NOS													65 (11.1)										65 (1.4)
BLADDER NECK											2 (0.6)												2 (0.0)
BLADDER, LATERAL WALL											5 (1.4)												5 (0.1)
BLADDER, NOS											57 (15.7)												57 (1.3)
BLADDER, OVERLAPPING LESION											11 (3.0)												11 (0.2)
BLADDER, POSTERIOR WALL											1 (0.3)												1 (0.0)
BODY OF PANCREAS																		7 (12.5)					7 (0.2)
BODY OF STOMACH										3 (7.9)													3 (0.1)
BONE MARROW														215 (57.3)									215 (4.8)
BONES-SKULL/FACE/ASSOC.JOINTS																	2 (2.3)						2 (0.0)
BORDER OF TONGUE													1 (0.2)										1 (0.0)
BRAIN, OVERLAPPING LESION					3 (8.3)																		3 (0.1)
BREAST, AXILLARY TAIL				3 (0.4)																			3 (0.1)
BREAST, CENTRAL PORTION				7 (0.9)																			7 (0.2)
BREAST, LOWER-INNER QUADRANT				11 (1.4)																			11 (0.2)
BREAST, LOWER-OUTER QUADRANT				20 (2.5)																			20 (0.4)
BREAST, NOS				37 (4.7)																			37 (0.8)
BREAST, NOS (EXCLUDES SKIN)				100 (12.7)																			100 (2.2)
BREAST, OVERLAPPING LESION				212 (26.8)																			212 (4.7)
BREAST, UPPER-INNER QUADRANT				32 (4.1)																			32 (0.7)
BREAST, UPPER-OUTER QUADRANT				80 (10.1)																			80 (1.8)
CARDIA, NOS								21 (17.1)		8 (21.1)													29 (0.6)
CECUM						11 (10.7)																	11 (0.2)

© 2024 Cinciripini PM et al. JAMA Oncology.

	Abdomen	Anal	Biliary	Breast	CNS	Colon	Endocrine	Esophagus	Eye/ear	GI	GU	Gyn	H/N	Heme	Liver	Lung	MSK	Pancreas	Prostate	Rectum	Skin	Unknown	All
	(N=26)	(N=33)	(N=7)	(N=790)	(N=36)	(N=103)	(N=105)	(N=123)	(N=20)	(N=38)	(N=363)	(N=203)	(N=587)	(N=375)	(N=43)	(N=782)	(N=86)	(N=56)	(N=285)	(N=151)	(N=301)	(N=13)	(N=4,526)
TR ICD-O Site Description	n (%)	n (%)	n (%)	n (%)	n (%)	n (%)	n (%)	n (%)	n (%)	n (%)	n (%)	n (%)	n (%)	n (%)	n (%)	n (%)	n (%)	n (%)	n (%)	n (%)	n (%)	n (%)	n (%)
CENTRAL PORTION OF BREAST				10 (1.3)																			10 (0.2)
CEREBELLUM, NOS					2 (5.6)																		2 (0.0)
CEREBRAL MENINGES					1 (2.8)																		1 (0.0)
CEREBRUM					7 (19.4)																		7 (0.2)
CERVICAL ESOPHAGUS								1 (0.8)															1 (0.0)
CERVIX UTERI												71 (35.0)											71 (1.6)
CERVIX UTERI, OVERLAPPING LESION												3 (1.5)											3 (0.1)
CHEEK MUCOSA													12 (2.0)										12 (0.3)
CHOROID									6 (30.0)														6 (0.1)
CILIARY BODY									6 (30.0)														6 (0.1)
CLITORIS												2 (1.0)											2 (0.0)
COLON, ASCENDING						9 (8.7)																	9 (0.2)
COLON, DESCENDING						6 (5.8)																	6 (0.1)
COLON, NOS						2 (1.9)																	2 (0.0)
COLON, SIGMOID						7 (6.8)																	7 (0.2)
COLON, SPLENIC FLEXURE						1 (1.0)																	1 (0.0)
COLON, TRANSVERSE						5 (4.9)																	5 (0.1)
CONJUNCTIVA									2 (10.0)														2 (0.0)
ONN/SUBCUT/SFT TISS,ABDOMEN																	1 (1.2)						1 (0.0)
CONN/SUBCUT/SFT TISS,HEAD/FACE/NECK																	2 (2.3)						2 (0.0)
CONN/SUBCUT/SFT TISS,LOW.LIMB/HIP																	21 (24.4)						21 (0.5)
CONN/SUBCUT/SFT TISS,PELVIS																	2 (2.3)						2 (0.0)
CONN/SUBCUT/SFT TISS,THORAX																	7 (8.1)						7 (0.2)
CONN/SUBCUT/SFT TISS,TRUNK, NOS																	2 (2.3)						2 (0.0)
CONN/SUBCUT/SFT TISS,UPP.LIMB/SHLDR																	9 (10.5)						9 (0.2)
CONNECTIVE, SUBQ, OTHER SOFT TISS; ABDOMEN																	1 (1.2)						1 (0.0)
CONNECTIVE, SUBQ, OTHER SOFT TISS; HEAD, FACE, NECK																	1 (1.2)						1 (0.0)
CONNECTIVE, SUBQ, OTHER SOFT TISS; LOWER LIMB, HIP																	8 (9.3)						8 (0.2)
CONNECTIVE, SUBQ, OTHER SOFT TISS; THORAX																	1 (1.2)						1 (0.0)
CONNECTIVE, SUBQ, OTHER SOFT TISS; UPPER LIMB, SHOULDER																	3 (3.5)						3 (0.1)
CORPUS UTERI												3 (1.5)											3 (0.1)
CORPUS UTERI, OVERLAPPING LESION												1 (0.5)											1 (0.0)
DESCENDING COLON						4 (3.9)																	4 (0.1)
DOME OF BLADDER											5 (1.4)												5 (0.1)
DORSAL SURFACE OF TONGUE, NOS													1 (0.2)										1 (0.0)
DUODENUM										3 (7.9)													3 (0.1)
ENDOCERVIX												5 (2.5)											5 (0.1)
ENDOMETRIUM												42 (20.7)											42 (0.9)
ESOPHAGUS, ABDOMINAL								1 (0.8)															1 (0.0)
ESOPHAGUS, CERVICAL								1 (0.8)															1 (0.0)
ESOPHAGUS, LOWER THIRD								16 (13.0)															16 (0.4)
ESOPHAGUS, MIDDLE THIRD								1 (0.8)															1 (0.0)
ESOPHAGUS, NOS								4 (3.3)															4 (0.1)

© 2024 Cinciripini PM et al. JAMA Oncology.

	Abdomen	Anal	Biliary	Breast	CNS	Colon	Endocrine	Esophagus	Eye/ear	GI	GU	Gyn	H/N	Heme	Liver	Lung	MSK	Pancreas	Prostate	Rectum	Skin	Unknown	All
	(N=26)	(N=33)	(N=7)	(N=790)	(N=36)	(N=103)	(N=105)	(N=123)	(N=20)	(N=38)	(N=363)	(N=203)	(N=587)	(N=375)	(N=43)	(N=782)	(N=86)	(N=56)	(N=285)	(N=151)	(N=301)	(N=13)	(N=4,526)
TR ICD-O Site Description	n (%)	n (%)	n (%)	n (%)	n (%)	n (%)	n (%)	n (%)	n (%)	n (%)	n (%)	n (%)	n (%)	n (%)	n (%)	n (%)	n (%)	n (%)	n (%)	n (%)	n (%)	n (%)	n (%)
ESOPHAGUS, OVERLAPPING LESION								1 (0.8)															1 (0.0)
ESOPHAGUS, UPPER THIRD								1 (0.8)															1 (0.0)
ETHMOID SINUS													1 (0.2)										1 (0.0)
EXTERNAL EAR									1 (5.0)												9 (3.0)		10 (0.2)
EXTERNAL LOWER LIP													3 (0.5)										3 (0.1)
EXTRAHEPATIC BILE DUCT			3 (42.9)																				3 (0.1)
EYE, NOS									2 (10.0)														2 (0.0)
EYELID																					8 (2.7)		8 (0.2)
FALLOPIAN TUBE												3 (1.5)											3 (0.1)
FEMALE BREAST, NOS				2 (0.3)																			2 (0.0)
FLOOR OF MOUTH, ANTERIOR													3 (0.5)										3 (0.1)
FLOOR OF MOUTH, LATERAL													1 (0.2)										1 (0.0)
FLOOR OF MOUTH, NOS													19 (3.2)										19 (0.4)
FLOOR OF MOUTH, OVERLAPPING LESION													1 (0.2)										1 (0.0)
FRONTAL LOBE					12 (33.3)																		12 (0.3)
FUNDUS OF STOMACH										1 (2.6)													1 (0.0)
GALLBLADDER			4 (57.1)																				4 (0.1)
GASTRIC ANTRUM										5 (13.2)													5 (0.1)
GLOTTIS													61 (10.4)										61 (1.3)
GUM, LOWER													5 (0.9)										5 (0.1)
GUM, NOS													1 (0.2)										1 (0.0)
HARD PALATE													4 (0.7)										4 (0.1)
HEAD OF PANCREAS																		16 (28.6)					16 (0.4)
HEPATIC FLEXURE OF COLON						2 (1.9)																	2 (0.0)
HYPOPHARYN.ASP.-ARYEPIGLOTTIC FOLD													5 (0.9)										5 (0.1)
HYPOPHARYNX, NOS													4 (0.7)										4 (0.1)
ILEUM (EXCLUDES ILEOCECAL VALVE)										1 (2.6)													1 (0.0)
INNER BREAST				2 (0.3)																			2 (0.0)
INTRAHEPATIC BILE DUCT															9 (20.9)								9 (0.2)
JEJUNUM										1 (2.6)													1 (0.0)
KIDNEY, NOS											201 (55.4)												201 (4.4)
LABIUM MAJUS																					2 (0.7)		2 (0.0)
LABIUM MINUS												2 (1.0)									1 (0.3)		3 (0.1)
LACRIMAL GLAND									2 (10.0)														2 (0.0)
LARYNX, NOS													8 (1.4)										8 (0.2)
LATERAL WALL OF BLADDER											7 (1.9)												7 (0.2)
LATERAL FLOOR OF MOUTH													1 (0.2)										1 (0.0)
LATERAL WALL OF OROPHARYNX													1 (0.2)										1 (0.0)
LATERAL WALL OF URINARY BLADDER											1 (0.3)												1 (0.0)
LESSER CURVATURE OF STOMACH, NOS										4 (10.5)													4 (0.1)
LIP, MUCOSA, NOS													1 (0.2)										1 (0.0)
LIVER															34 (79.1)								34 (0.8)
LONG BONES-LOW.LIMB/ASSOC.JOINTS																	5 (5.8)						5 (0.1)

© 2024 Cinciripini PM et al. JAMA Oncology.

	Abdomen	Anal	Biliary	Breast	CNS	Colon	Endocrine	Esophagus	Eye/ear	GI	GU	Gyn	H/N	Heme	Liver	Lung	MSK	Pancreas	Prostate	Rectum	Skin	Unknown	All
	(N=26)	(N=33)	(N=7)	(N=790)	(N=36)	(N=103)	(N=105)	(N=123)	(N=20)	(N=38)	(N=363)	(N=203)	(N=587)	(N=375)	(N=43)	(N=782)	(N=86)	(N=56)	(N=285)	(N=151)	(N=301)	(N=13)	(N=4,526)
TR ICD-O Site Description	n (%)	n (%)	n (%)	n (%)	n (%)	n (%)	n (%)	n (%)	n (%)	n (%)	n (%)	n (%)	n (%)	n (%)	n (%)	n (%)	n (%)	n (%)	n (%)	n (%)	n (%)	n (%)	n (%)
LONG BONES-UPP.LIMB/SCAPULA/AS.JNT																	1 (1.2)						1 (0.0)
LONG BONES OF UPPER LIMB, SCAPULA																	1 (1.2)						1 (0.0)
LOWER GUM													10 (1.7)										10 (0.2)
LOWER LIMB AND ASSOCIATED JOINTS, LONG BONES																	1 (1.2)						1 (0.0)
LOWER LIP, EXTERNAL													3 (0.5)										3 (0.1)
LOWER LIP, MUCOSA													2 (0.3)										2 (0.0)
LOWER LOBE, LUNG																169 (21.6)							169 (3.7)
LOWER THIRD OF ESOPHAGUS								59 (48.0)															59 (1.3)
LOWER-INNER QUADRANT OF BREAST				18 (2.3)																			18 (0.4)
LOWER-OUTER QUADRANT OF BREAST				23 (2.9)																			23 (0.5)
LUNG, LOWER LOBE																29 (3.7)							29 (0.6)
LUNG, MIDDLE LOBE																5 (0.6)							5 (0.1)
LUNG, NOS																23 (2.9)							23 (0.5)
LUNG, OVERLAPPING LESION																5 (0.6)							5 (0.1)
LUNG, UPPER LOBE																101 (12.9)							101 (2.2)
LYMPH NODE, NOS														141 (37.6)									141 (3.1)
LYMPH NODES OF HEAD, FACE AND NECK														1 (0.3)									1 (0.0)
LYMPH NODES OF MULTIPLE REGIONS														7 (1.9)									7 (0.2)
LYMPH NODES, INTRATHORACIC														4 (1.1)									4 (0.1)
LYMPH NODES, MULTIPLE REGIONS														3 (0.8)									3 (0.1)
LYMPH NODES, PELVIC														1 (0.3)									1 (0.0)
MAIN BRONCHUS																14 (1.8)							14 (0.3)
MANDIBLE													1 (0.2)										1 (0.0)
MAXILLARY SINUS													5 (0.9)										5 (0.1)
MIDDLE LOBE, LUNG																25 (3.2)							25 (0.6)
MIDDLE THIRD OF ESOPHAGUS								9 (7.3)															9 (0.2)
MOUTH, OVERLAPPING LESION													1 (0.2)										1 (0.0)
NASAL CAVITY													4 (0.7)				1 (1.2)						5 (0.1)
NASAL CAVITY (EXCLUDES NOSE, NOS)													19 (3.2)										19 (0.4)
NASOPHARYNX, LATERAL WALL													1 (0.2)										1 (0.0)
NASOPHARYNX, NOS													12 (2.0)										12 (0.3)
NIPPLE				2 (0.3)																			2 (0.0)
NOT AVAILABLE OR NOT APPLICABLE									1 (5.0)				1 (0.2)				2 (2.3)						4 (0.1)
OROPHARYNX, LATERAL WALL													1 (0.2)										1 (0.0)
OROPHARYNX, NOS													12 (2.0)										12 (0.3)
OROPHARYNX, OVERLAPPING LESION													2 (0.3)										2 (0.0)
OTHER PARTS OF URINARY BLADDER											1 (0.3)												1 (0.0)
OVARY												42 (20.7)											42 (0.9)
PALATE, HARD													1 (0.2)										1 (0.0)
PALATE, NOS													1 (0.2)										1 (0.0)
PALATE, OVERLAPPING LESION													1 (0.2)										1 (0.0)
PALATE, SOFT, NOS													1 (0.2)										1 (0.0)
PANCREAS, BODY																		3 (5.4)					3 (0.1)

© 2024 Cinciripini PM et al. JAMA Oncology.

	Abdomen	Anal	Biliary	Breast	CNS	Colon	Endocrine	Esophagus	Eye/ear	GI	GU	Gyn	H/N	Heme	Liver	Lung	MSK	Pancreas	Prostate	Rectum	Skin	Unknown	All
	(N=26)	(N=33)	(N=7)	(N=790)	(N=36)	(N=103)	(N=105)	(N=123)	(N=20)	(N=38)	(N=363)	(N=203)	(N=587)	(N=375)	(N=43)	(N=782)	(N=86)	(N=56)	(N=285)	(N=151)	(N=301)	(N=13)	(N=4,526)
TR ICD-O Site Description	n (%)	n (%)	n (%)	n (%)	n (%)	n (%)	n (%)	n (%)	n (%)	n (%)	n (%)	n (%)	n (%)	n (%)	n (%)	n (%)	n (%)	n (%)	n (%)	n (%)	n (%)	n (%)	n (%)
PANCREAS, HEAD																		9 (16.1)					9 (0.2)
PANCREAS, NOS																		4 (7.1)					4 (0.1)
PANCREAS, OTHER PARTS																		1 (1.8)					1 (0.0)
PANCREAS, OVERLAPPING LESION																		8 (14.3)					8 (0.2)
PANCREAS, TAIL																		1 (1.8)					1 (0.0)
PARATHYROID GLAND							1 (1.0)																1 (0.0)
PARIETAL LOBE					6 (16.7)																		6 (0.1)
PAROTID GLAND													12 (2.0)										12 (0.3)
PELV BONES/SACRUM/COCCYX/ASSOC.JNTS																	8 (9.3)						8 (0.2)
PENIS, NOS											2 (0.6)										3 (1.0)		5 (0.1)
PERITONEUM, NOS	9 (34.6)																						9 (0.2)
PHARYNX, NOS													4 (0.7)										4 (0.1)
PLACENTA												2 (1.0)											2 (0.0)
PLEURA, NOS																2 (0.3)							2 (0.0)
POSTERIOR WALL OF BLADDER											9 (2.5)												9 (0.2)
POSTERIOR WALL OF OROPHARYNX													3 (0.5)										3 (0.1)
POSTERIOR WALL OF URINARY BLADDER											1 (0.3)												1 (0.0)
PROSTATE GLAND																			285 (100.0)				285 (6.3)
PYRIFORM SINUS													11 (1.9)										11 (0.2)
RECTOSIGMOID JUNCTION						1 (1.0)														30 (19.9)			31 (0.7)
RECTUM, ANUS AND ANAL CANAL, OVERLAPPING LESION		1 (3.0)																					1 (0.0)
RECTUM, NOS																				117 (77.5)			117 (2.6)
RENAL PELVIS											13 (3.6)												13 (0.3)
RETICULOENDOTHELIAL SYSTEM, NOS																	1 (1.2)						1 (0.0)
RETROMOLAR AREA													11 (1.9)										11 (0.2)
RETROPERITONEUM	16 (61.5)																						16 (0.4)
RIB, STERNUM, CLAVICLE AND ASSOCIATED JOINTS																	1 (1.2)						1 (0.0)
SCROTUM, NOS																					2 (0.7)		2 (0.0)
SIGMOID COLON						22 (21.4)																	22 (0.5)
SKIN OF ARM AND SHOULDER																					2 (0.7)		2 (0.0)
SKIN OF LEG AND HIP																					1 (0.3)		1 (0.0)
SKIN OF LIP, NOS																					3 (1.0)		3 (0.1)
SKIN OF LOWER LIMB																					27 (9.0)		27 (0.6)
SKIN OF LOWER LIMB AND HIP																					11 (3.7)		11 (0.2)
SKIN OF OTHER AND UNSPECIFIED PARTS OF FACE																					10 (3.3)		10 (0.2)
SKIN OF OTHER/UNSPEC.PARTS OF FACE																					51 (16.9)		51 (1.1)
SKIN OF SCALP AND NECK																					27 (9.0)		27 (0.6)
SKIN OF TRUNK																					77 (25.6)		77 (1.7)
SKIN OF UPPER LIMB AND SHOULDER																					12 (4.0)		12 (0.3)
SKIN OF UPPER LIMB/SHOULDER																					20 (6.6)		20 (0.4)
SKIN, NOS																	1 (1.2)						1 (0.0)
SKIN, NOS (EXCLUDES SKIN OF VULVA)																					30 (10.0)		30 (0.7)
SKIN, OTHER & UNSPEC PARTS OF FACE																					1 (0.3)		1 (0.0)

© 2024 Cinciripini PM et al. JAMA Oncology.

	Abdomen	Anal	Biliary	Breast	CNS	Colon	Endocrine	Esophagus	Eye/ear	GI	GU	Gyn	H/N	Heme	Liver	Lung	MSK	Pancreas	Prostate	Rectum	Skin	Unknown	All
	(N=26)	(N=33)	(N=7)	(N=790)	(N=36)	(N=103)	(N=105)	(N=123)	(N=20)	(N=38)	(N=363)	(N=203)	(N=587)	(N=375)	(N=43)	(N=782)	(N=86)	(N=56)	(N=285)	(N=151)	(N=301)	(N=13)	(N=4,526)
TR ICD-O Site Description	n (%)	n (%)	n (%)	n (%)	n (%)	n (%)	n (%)	n (%)	n (%)	n (%)	n (%)	n (%)	n (%)	n (%)	n (%)	n (%)	n (%)	n (%)	n (%)	n (%)	n (%)	n (%)	n (%)
SKIN, UNSPECIFIED MALIGNANT NEOPLASM OF SKIN OF EAR AND EXTE																					2 (0.7)		2 (0.0)
SKIN, UNSPECIFIED MALIGNANT NEOPLASM OF SKIN OF EYELID, INCL																					1 (0.3)		1 (0.0)
SMALL INTESTINE, NOS										2 (5.3)													2 (0.0)
SOFT PALATE, NOS													1 (0.2)										1 (0.0)
SOFT PALATE,NOS (EXCL NASOPHARYNGL)													10 (1.7)										10 (0.2)
SOFT TISSUES OF HEAD, FACE, & NECK																	1 (1.2)						1 (0.0)
SOFT TISSUES OF UPP. LIMB, SHOULDER																	1 (1.2)						1 (0.0)
SPECIFIED PARTS OF PERITONEUM	1 (3.8)																						1 (0.0)
SPLEEN														1 (0.3)									1 (0.0)
STOMACH, GREATER CURVATURE, NOS										2 (5.3)													2 (0.0)
STOMACH, NOS										5 (13.2)													5 (0.1)
STOMACH, OVERLAPPING LESION										3 (7.9)													3 (0.1)
SUBGLOTTIS													1 (0.2)										1 (0.0)
SUBMANDIBULAR GLAND													4 (0.7)										4 (0.1)
SUPRAGLOTTIS													67 (11.4)										67 (1.5)
TAIL OF PANCREAS																		3 (5.4)					3 (0.1)
TEMPORAL LOBE					4 (11.1)																		4 (0.1)
TESTIS, NOS											27 (7.4)												27 (0.6)
THORACIC ESOPHAGUS								5 (4.1)															5 (0.1)
THYMUS														2 (0.5)									2 (0.0)
THYROID GLAND							99 (94.3)																99 (2.2)
TONGUE, ANT 2/3, NOS													12 (2.0)										12 (0.3)
TONGUE, BORDER													2 (0.3)										2 (0.0)
TONGUE, NOS													4 (0.7)										4 (0.1)
TONGUE, OVERLAPPING LESION													1 (0.2)										1 (0.0)
TONGUE, VENTRAL SURFACE, NOS													4 (0.7)										4 (0.1)
TONSIL, NOS													39 (6.6)										39 (0.9)
TONSILLAR FOSSA													40 (6.8)										40 (0.9)
TONSILLAR PILLAR													6 (1.0)										6 (0.1)
TRACHEA																2 (0.3)							2 (0.0)
TRANSVERSE COLON						11 (10.7)																	11 (0.2)
TRIGONE OF BLADDER											6 (1.7)												6 (0.1)
UNKNOWN PRIMARY SITE																						13 (100.0)	13 (0.3)
UPPER LIMB, SCAPULA & ASSOCIATED JOINTS, LONG BONES																	1 (1.2)						1 (0.0)
UPPER LOBE, LUNG																400 (51.2)							400 (8.8)
UPPER THIRD OF ESOPHAGUS								3 (2.4)															3 (0.1)
UPPER-INNER QUADRANT OF BREAST				67 (8.5)																			67 (1.5)
UPPER-OUTER QUADRANT OF BREAST				160 (20.3)																			160 (3.5)
URETER											3 (0.8)												3 (0.1)
URETHRA											7 (1.9)												7 (0.2)
URINARY BLADDER, NOS											1 (0.3)												1 (0.0)
UTERUS, NOS												1 (0.5)											1 (0.0)
UVULA													2 (0.3)										2 (0.0)

© 2024 Cinciripini PM et al. JAMA Oncology.

	Abdomen	Anal	Biliary	Breast	CNS	Colon	Endocrine	Esophagus	Eye/ear	GI	GU	Gyn	H/N	Heme	Liver	Lung	MSK	Pancreas	Prostate	Rectum	Skin	Unknown	All
	(N=26)	(N=33)	(N=7)	(N=790)	(N=36)	(N=103)	(N=105)	(N=123)	(N=20)	(N=38)	(N=363)	(N=203)	(N=587)	(N=375)	(N=43)	(N=782)	(N=86)	(N=56)	(N=285)	(N=151)	(N=301)	(N=13)	(N=4,526)
**TR ICD-O Site Description**	n (%)	n (%)	n (%)	n (%)	n (%)	n (%)	n (%)	n (%)	n (%)	n (%)	n (%)	n (%)	n (%)	n (%)	n (%)	n (%)	n (%)	n (%)	n (%)	n (%)	n (%)	n (%)	n (%)
**VAGINA, NOS**												7 (3.4)											7 (0.2)
**VALLECULA**													1 (0.2)										1 (0.0)
**VENTRAL SURFACE OF TONGUE, NOS**													13 (2.2)										13 (0.3)
**VENTRICLE, NOS**					1 (2.8)																		1 (0.0)
**VERTEBRAL COLUMN(EXCL.SACRUM/COCCYX**																	1 (1.2)						1 (0.0)
**VULVA, NOS**												19 (9.4)									1 (0.3)		20 (0.4)

© 2024 Cinciripini PM et al. JAMA Oncology.

eTable 2. 3-, 6- and 9-month abstinence rates for all patients with staging and by time of entry into the TRTP for the patients with stage (n=4526).

	**All Patients** (N=4,526)	**< 6 mos** (N=2,143)	**6 mos - 5 yrs** (N=1,703)	**5 yrs +** (N=680)
**Intention-to-Treat**	N (%)	N (%)	N (%)	N (%)
3 month				
non-abstinent	2,626 (58.0)	1,076 (50.2)	1,117 (65.6)	433 (63.7)
abstinent	1,900 (42.0)	1,067 (49.8)	586 (34.4)	247 (36.3)
6 month				
non-abstinent	2,715 (60.0)	1,049 (50.7)	1,096 (66.5)	436 (64.6)
abstinent	1,811 (40.0)	1,019 (49.3)	553 (33.5)	239 (35.4)
9 month				
non-abstinent	2,891 (63.9)	1,065 (53.4)	1,080 (68.4)	452 (68.5)
abstinent	1,635 (36.1)	928 (46.6)	499 (31.6)	208 (31.5)
**Respondent Only**				
3 month				
non-abstinent	2,109 (52.6)	822 (43.5)	920 (61.1)	367 (59.8)
abstinent	1,900 (47.4)	1,067 (56.5)	586 (38.9)	247 (40.2)
6 month				
non-abstinent	1,802 (49.9)	695 (40.5)	800 (59.1)	307 (56.2)
abstinent	1,811 (50.1)	1,019 (59.5)	553 (40.9)	239 (43.8)
9 month				
non-abstinent	1,620 (49.8)	617 (39.9)	725 (59.2)	278 (57.2)
abstinent	1,635 (50.2)	928 (60.1)	499 (40.8)	208 (42.8)

© 2024 Cinciripini PM et al. JAMA Oncology.

eTable 3. Patient characteristics for both staged and non-staged patients

Characteristic	All Patients (N=6,397)
Gender	N (%)
Female	3,161 (49.4)
Male	3,236 (50.6)
Race	
White	5,001 (78.2)
Black	713 (11.1)
Other	683 (10.7)
Disease Site	
Abdomen	35 (0.5)
Anal	51 (0.8)
Biliary	9 (0.1)
Breast	1,014 (15.9)
CNS	70 (1.1)
Colon	140 (2.2)
Endocrine	140 (2.2)
Esophagus	154 (2.4)
Eye/ear	34 (0.5)
GI	53 (0.8)
GU	513 (8.0)
Gyn	293 (4.6)
H/N	789 (12.3)
Heme	575 (9.0)
Liver	78 (1.2)
Lung	1,118 (17.5)
MSK	123 (1.9)
Pancreas	101 (1.6)
Prostate	377 (5.9)
Rectum	184 (2.9)
Skin	469 (7.3)
Unknown	
Stage	
I/II	2,133 (33.3)
III	1,063 (16.6)
IV	1,330 (20.8)
Missing	1,871 (29.2)
	55 (47-62)
Age at Diagnosis	23 (0-40)
Pack Yrs .	15 (9-20)

© 2024 Cinciripini PM et al. JAMA Oncology.

CPD	4 (3-6)
FTCD	19 (14-26)

Abbreviations: CNS = Central Nervous System;GI = Gastrointestinal; GU = Genitourinary; Gyn = Gynecologic; H/N = Head & Neck; Heme = Hematologic; MSK = Musculoskeletal. CPD = Cigarettes per day; FTCD, Fagerström Test for Cigarette Dependence.

Note: Abstinence values are at 3 months post-TRTP entry.

© 2024 Cinciripini PM et al. JAMA Oncology.

eTable 4. 3-, 6- and 9-month abstinence rates for all patients and time of entry into the TRTP for all (staged and non-staged patients, n= 6,397).

	**All Patients** (N=6,397)	**< 6 mos** (N=3,014)	**6 mos - 5 yrs** (N=2,373)	**5 yrs +** (N=1,010)
**Intention-to-Treat**	N (%)	N (%)	N (%)	N (%)
3 month				
non-abstinent	3,754 (58.7)	1,539 (51.1)	1,569 (66.1)	646 (64.0)
abstinent	2,643 (41.3)	1,475 (48.9)	804 (33.9)	364 (36.0)
6 month				
non-abstinent	3,882 (60.7)	1,610 (53.4)	1,611 (67.9)	661 (65.4)
abstinent	2,515 (39.3)	1,404 (46.6)	762 (32.1)	349 (34.6)
9 month				
non-abstinent	4,144 (64.8)	1,761 (58.4)	1,678 (70.7)	705 (69.8)
abstinent	2,253 (35.2)	1,253 (41.6)	695 (29.3)	305 (30.2)
**Respondent Only**				
3 month				
non-abstinent	3,019 (53.3)	1,194 (44.7)	1,286 (61.5)	539 (59.7)
abstinent	2,643 (46.7)	1,475 (55.3)	804 (38.5)	364 (40.3)
6 month				
non-abstinent	2,561 (50.5)	994 (41.5)	1,110 (59.3)	457 (56.7)
abstinent	2,515 (49.5)	1,404 (58.5)	762 (40.7)	349 (43.3)
9 month				
non-abstinent	2,283 (50.3)	887 (41.4)	986 (58.7)	410 (57.3)
abstinent	2,253 (49.7)	1,253 (58.6)	695 (41.3)	305 (42.7)

© 2024 Cinciripini PM et al. JAMA Oncology.

eTable 5. Difference in Abstinence Hazard Rates by Time for Cancer Dx to TRTP

Abstinent Hazard Rate - Non-Abstinent Hazard Rate

Months from Diagnosis to TRTP	*dy/dx*	*P-value*	*95% CI*	
1	-0.26	< 0.001	-0.35	-0.17
2	-0.25	< 0.001	-0.34	-0.17
3	-0.25	< 0.001	-0.33	-0.17
4	-0.24	< 0.001	-0.32	-0.16
5	-0.24	< 0.001	-0.31	-0.16
6	-0.23	< 0.001	-0.31	-0.16
7	-0.23	< 0.001	-0.30	-0.15
8	-0.22	< 0.001	-0.29	-0.15
9	-0.22	< 0.001	-0.29	-0.15
10	-0.21	< 0.001	-0.28	-0.14
11	-0.21	< 0.001	-0.27	-0.14
12	-0.20	< 0.001	-0.27	-0.13
13	-0.20	< 0.001	-0.26	-0.13
14	-0.19	< 0.001	-0.25	-0.13
15	-0.19	< 0.001	-0.25	-0.12
16	-0.18	< 0.001	-0.24	-0.12
17	-0.18	< 0.001	-0.24	-0.12
18	-0.17	< 0.001	-0.23	-0.11
19	-0.17	< 0.001	-0.23	-0.11
20	-0.16	< 0.001	-0.22	-0.10
21	-0.16	< 0.001	-0.22	-0.10
22	-0.16	< 0.001	-0.22	-0.10
23	-0.15	< 0.001	-0.21	-0.09
24	-0.15	< 0.001	-0.21	-0.09
25	-0.14	< 0.001	-0.20	-0.09
26	-0.14	< 0.001	-0.20	-0.08
27	-0.14	< 0.001	-0.20	-0.08
28	-0.13	< 0.001	-0.19	-0.07
29	-0.13	< 0.001	-0.19	-0.07
30	-0.13	< 0.001	-0.19	-0.07
31	-0.12	< 0.001	-0.18	-0.06
32	-0.12	< 0.001	-0.18	-0.06
33	-0.12	< 0.001	-0.18	-0.06
34	-0.11	< 0.001	-0.17	-0.05
35	-0.11	< 0.001	-0.17	-0.05
36	-0.11	0.001	-0.17	-0.05
37	-0.10	0.001	-0.17	-0.04
38	-0.10	0.001	-0.16	-0.04
39	-0.10	0.002	-0.16	-0.04
40	-0.10	0.003	-0.16	-0.03
41	-0.09	0.004	-0.16	-0.03
42	-0.09	0.005	-0.16	-0.03
43	-0.09	0.007	-0.15	-0.02

© 2024 Cinciripini PM et al. JAMA Oncology.

Abstinent Hazard Rate - Non-Abstinent Hazard Rate

Months from Diagnosis to TRTP	*dy/dx*	*P-value*	*95% CI*	
44	-0.09	0.009	-0.15	-0.02
45	-0.08	0.012	-0.15	-0.02
46	-0.08	0.016	-0.15	-0.02
47	-0.08	0.02	-0.14	-0.01
48	-0.08	0.024	-0.14	-0.01
49	-0.07	0.03	-0.14	-0.01
50	-0.07	0.036	-0.14	0.00
51	-0.07	0.043	-0.14	0.00
52	-0.07	0.051	-0.14	0.00
53	-0.07	0.06	-0.13	0.00
54	-0.06	0.07	-0.13	0.01
55	-0.06	0.081	-0.13	0.01
56	-0.06	0.092	-0.13	0.01
57	-0.06	0.105	-0.13	0.01
58	-0.06	0.118	-0.13	0.01
59	-0.05	0.132	-0.12	0.02
60	-0.05	0.147	-0.12	0.02
61	-0.05	0.162	-0.12	0.02
62	-0.05	0.178	-0.12	0.02
63	-0.05	0.195	-0.12	0.02
64	-0.05	0.212	-0.12	0.03
65	-0.04	0.23	-0.11	0.03
66	-0.04	0.248	-0.11	0.03
67	-0.04	0.267	-0.11	0.03
68	-0.04	0.286	-0.11	0.03
69	-0.04	0.305	-0.11	0.03
70	-0.04	0.324	-0.11	0.04
71	-0.03	0.344	-0.11	0.04
72	-0.03	0.364	-0.11	0.04
73	-0.03	0.384	-0.10	0.04
74	-0.03	0.404	-0.10	0.04
75	-0.03	0.424	-0.10	0.04
76	-0.03	0.444	-0.10	0.04
77	-0.03	0.464	-0.10	0.05
78	-0.03	0.484	-0.10	0.05
79	-0.02	0.503	-0.10	0.05
80	-0.02	0.523	-0.10	0.05
81	-0.02	0.543	-0.09	0.05
82	-0.02	0.562	-0.09	0.05
83	-0.02	0.581	-0.09	0.05
84	-0.02	0.6	-0.09	0.05
85	-0.02	0.619	-0.09	0.05
86	-0.02	0.638	-0.09	0.05
87	-0.02	0.656	-0.09	0.06
88	-0.02	0.674	-0.09	0.06

© 2024 Cinciripini PM et al. JAMA Oncology.

Abstinent Hazard Rate - Non-Abstinent Hazard Rate

Months from Diagnosis to TRTP	dy/dx	P-value	95% CI	
89	-0.01	0.692	-0.09	0.06
90	-0.01	0.709	-0.09	0.06
91	-0.01	0.727	-0.08	0.06
92	-0.01	0.744	-0.08	0.06
93	-0.01	0.76	-0.08	0.06
94	-0.01	0.777	-0.08	0.06
95	-0.01	0.793	-0.08	0.06
96	-0.01	0.809	-0.08	0.06
97	-0.01	0.824	-0.08	0.06
98	-0.01	0.839	-0.08	0.06
99	-0.01	0.855	-0.08	0.06
100	-0.01	0.869	-0.08	0.06
101	-0.01	0.884	-0.07	0.06
102	0.00	0.898	-0.07	0.06
103	0.00	0.912	-0.07	0.07
104	0.00	0.925	-0.07	0.07
105	0.00	0.939	-0.07	0.07
106	0.00	0.952	-0.07	0.07
107	0.00	0.965	-0.07	0.07
108	0.00	0.977	-0.07	0.07
109	0.00	0.99	-0.07	0.07
110	0.00	0.998	-0.07	0.07
111	0.00	0.987	-0.07	0.07
112	0.00	0.975	-0.07	0.07
113	0.00	0.964	-0.07	0.07
114	0.00	0.953	-0.06	0.07
115	0.00	0.942	-0.06	0.07
116	0.00	0.931	-0.06	0.07
117	0.00	0.921	-0.06	0.07
118	0.00	0.91	-0.06	0.07
119	0.00	0.9	-0.06	0.07
120	0.00	0.89	-0.06	0.07
121	0.00	0.881	-0.06	0.07
122	0.01	0.871	-0.06	0.07
123	0.01	0.862	-0.06	0.07
124	0.01	0.853	-0.06	0.07
125	0.01	0.844	-0.06	0.07
126	0.01	0.836	-0.06	0.07
127	0.01	0.827	-0.06	0.07
128	0.01	0.819	-0.06	0.07
129	0.01	0.811	-0.05	0.07
130	0.01	0.803	-0.05	0.07
131	0.01	0.795	-0.05	0.07
132	0.01	0.787	-0.05	0.07
133	0.01	0.78	-0.05	0.07

© 2024 Cinciripini PM et al. JAMA Oncology.

Abstinent Hazard Rate - Non-Abstinent Hazard Rate

Months from Diagnosis to TRTP	*dy/dx*	*P-value*	*95% CI*	
134	0.01	0.772	-0.05	0.07
135	0.01	0.765	-0.05	0.07
136	0.01	0.758	-0.05	0.07
137	0.01	0.751	-0.05	0.07
138	0.01	0.744	-0.05	0.07
139	0.01	0.738	-0.05	0.07
140	0.01	0.731	-0.05	0.07
141	0.01	0.725	-0.05	0.07
142	0.01	0.718	-0.05	0.07
143	0.01	0.712	-0.05	0.07
144	0.01	0.706	-0.05	0.07
145	0.01	0.7	-0.05	0.07
146	0.01	0.694	-0.05	0.07
147	0.01	0.689	-0.04	0.07
148	0.01	0.683	-0.04	0.07
149	0.01	0.678	-0.04	0.07
150	0.01	0.672	-0.04	0.07
151	0.01	0.667	-0.04	0.07
152	0.01	0.662	-0.04	0.07
153	0.01	0.657	-0.04	0.07
154	0.01	0.652	-0.04	0.07
155	0.01	0.647	-0.04	0.07
156	0.01	0.642	-0.04	0.07
157	0.01	0.637	-0.04	0.07
158	0.01	0.633	-0.04	0.06
159	0.01	0.628	-0.04	0.06
160	0.01	0.624	-0.04	0.06
161	0.01	0.619	-0.04	0.06
162	0.01	0.615	-0.04	0.06
163	0.01	0.611	-0.04	0.06
164	0.01	0.607	-0.04	0.06
165	0.01	0.603	-0.04	0.06
166	0.01	0.599	-0.04	0.06
167	0.01	0.595	-0.04	0.06
168	0.01	0.591	-0.04	0.06
169	0.01	0.587	-0.04	0.06
170	0.01	0.583	-0.03	0.06
171	0.01	0.58	-0.03	0.06
172	0.01	0.576	-0.03	0.06
173	0.01	0.572	-0.03	0.06
174	0.01	0.569	-0.03	0.06
175	0.01	0.566	-0.03	0.06
176	0.01	0.562	-0.03	0.06
177	0.01	0.559	-0.03	0.06
178	0.01	0.556	-0.03	0.06

© 2024 Cinciripini PM et al. JAMA Oncology.

Abstinent Hazard Rate - Non-Abstinent Hazard Rate

Months from Diagnosis to TRTP	*dy/dx*	*P-value*	*95% CI*	
179	0.01	0.553	-0.03	0.06
180	0.01	0.549	-0.03	0.06
181	0.01	0.546	-0.03	0.06
182	0.01	0.543	-0.03	0.06

© 2024 Cinciripini PM et al. JAMA Oncology.

eTable 6. Multivariate Analyses for Survival According to Cancer Diagnosis and Time between Diagnosis and Entry into the TRTP for the Primary Cohort of Patients with Stage (N=4,426) Using Multiple Imputation.

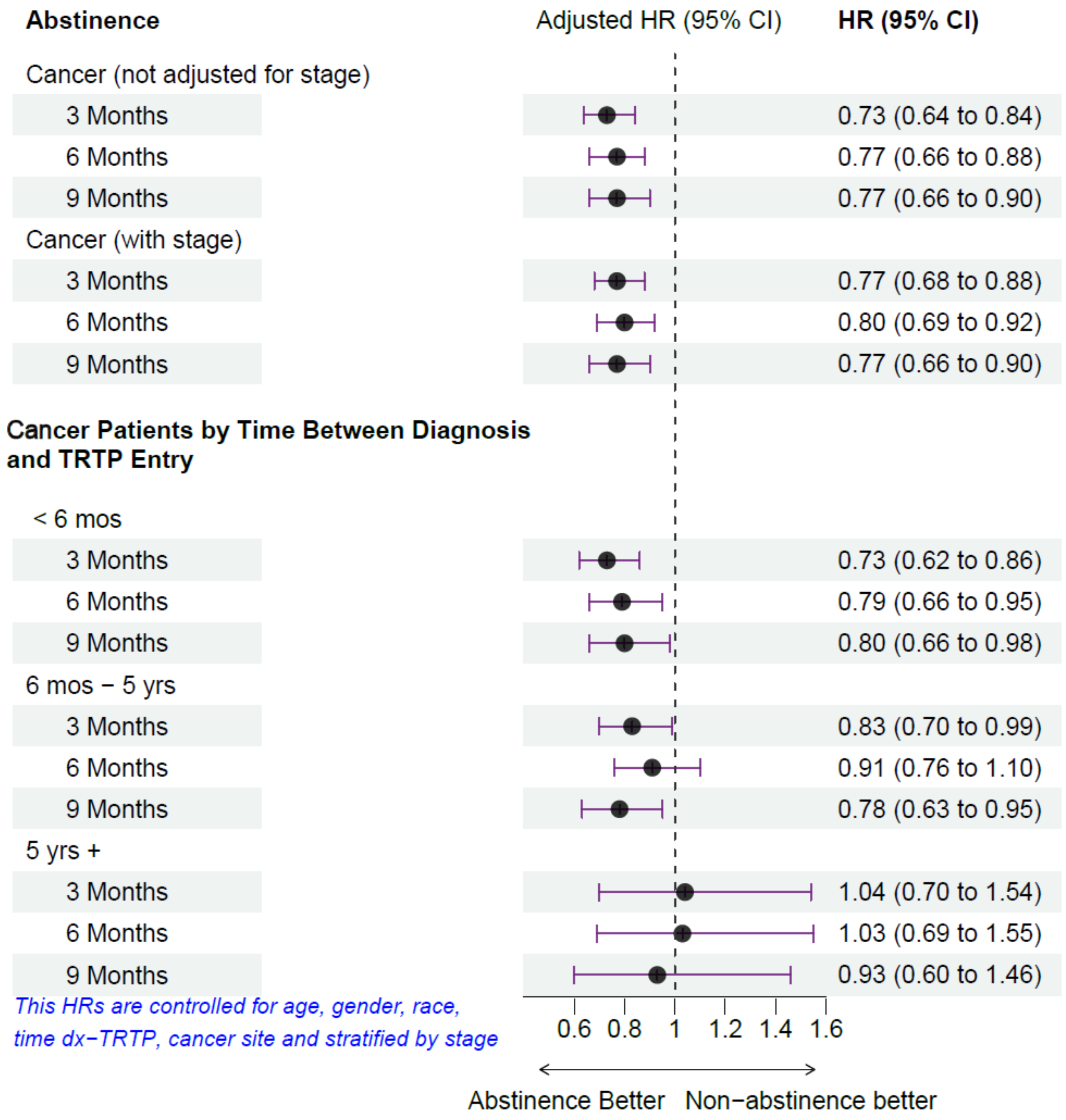

© 2024 Cinciripini PM et al. JAMA Oncology.

eTable 7. Multivariate analyses for survival according to cancer diagnosis and time between diagnosis and entry into the TRTP for the pooled sample (staged and non-staged patients (n=6,397)).

	No. of Deaths / No. of Patients (%)		
**Abstinence**	**Non-Abstinent**	**Abstinent**	**HR (95% CI)**
Cancer (not adjusted for stage)			
3 Months	1276/3754 (33.99)	791/2643 (29.93)	0.77 (0.71 to 0.85)
6 Months	1166/3696 (31.55)	713/2513 (28.37)	0.81 (0.73 to 0.88)
9 Months	1061/3738 (28.38)	594/2247 (26.44)	0.82 (0.74 to 0.90)
Cancer (stratified by stage)			
3 Months	1276/3754 (33.99)	791/2643 (29.93)	0.80 (0.73 to 0.87)
6 Months	1166/3696 (31.55)	713/2513 (28.37)	0.81 (0.74 to 0.89)
9 Months	1061/3738 (28.38)	594/2247 (26.44)	0.81 (0.73 to 0.90)
**Cancer Patients by Time Between Diagnosis and TRTP Entry**			
< 6 mos			
3 Months	619/1539 (40.22)	491/1475 (33.29)	0.76 (0.67 to 0.86)
6 Months	561/1503 (37.33)	440/1402 (31.38)	0.77 (0.68 to 0.88)
9 Months	521/1546 (33.70)	369/1248 (29.57)	0.78 (0.68 to 0.89)
6 mos - 5 yrs			
3 Months	435/1117 (38.94)	193/586 (32.94)	0.81 (0.70 to 0.94)
6 Months	506/1538 (32.90)	219/762 (28.74)	0.83 (0.71 to 0.97)
9 Months	446/1511 (29.52)	185/695 (26.62)	0.82 (0.69 to 0.98)
5 yrs +			
3 Months	104/646 (16.10)	55/364 (15.11)	0.96 (0.69 to 1.34)
6 Months	99/655 (15.11)	54/349 (15.47)	0.96 (0.68 to 1.34)
9 Months	94/681 (13.80)	40/304 (13.16)	0.89 (0.61 to 1.31)

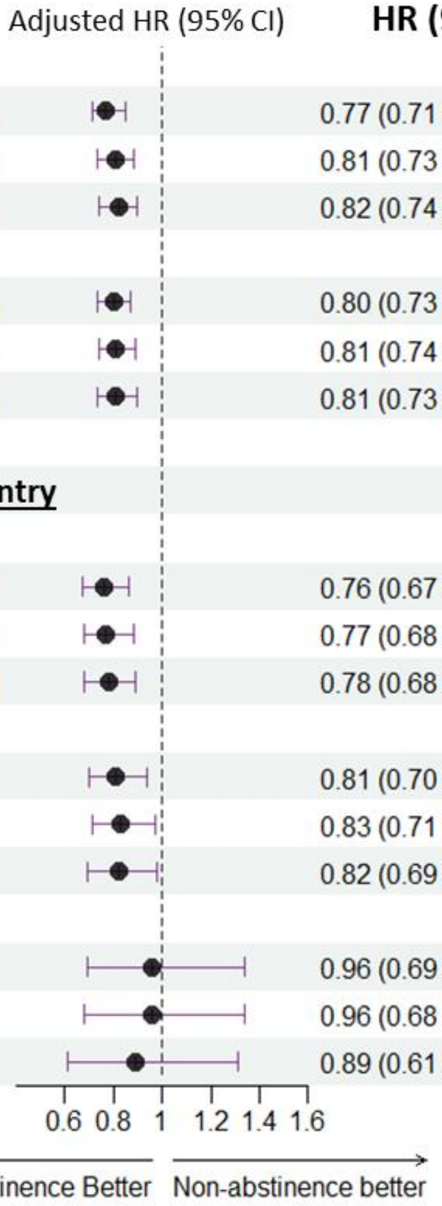

This HRs are controlled for age, gender, race, time dx-TTP site and stratified by stage

© 2024 Cinciripini PM et al. JAMA Oncology.

eFigure. Difference in abstinence hazard rates by time from diagnosis to TRTP by cohort

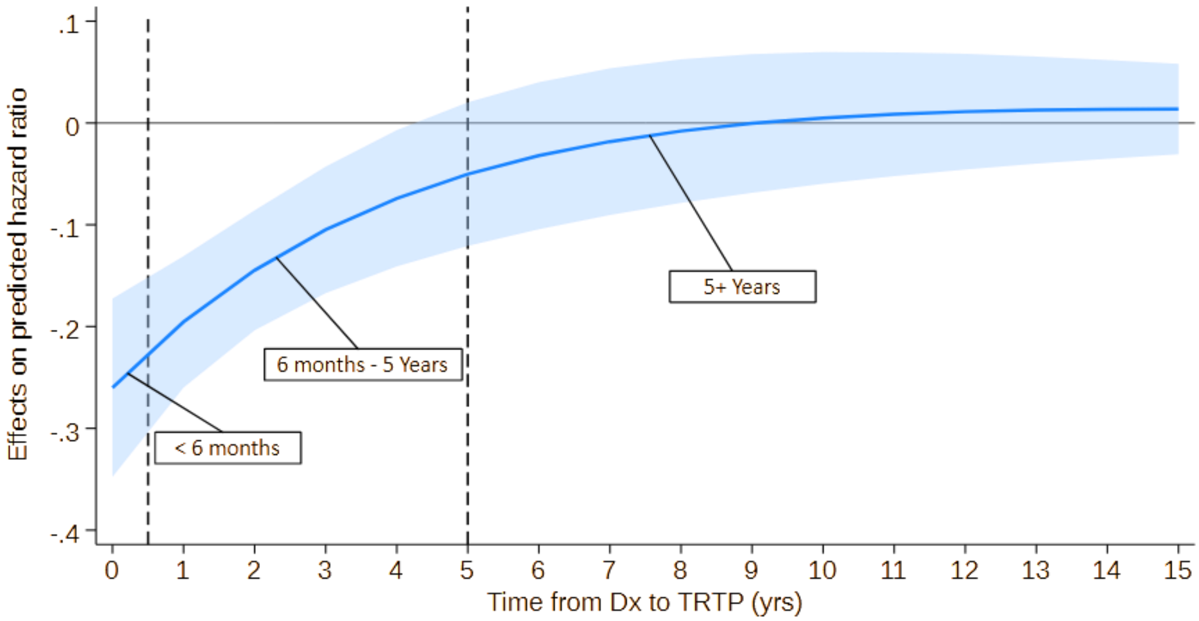

© 2024 Cinciripini PM et al. JAMA Oncology.

eReferences

1. Karam-Hage M, Kypriotakis G, Robinson JD, et al. Improvement of Smoking Abstinence Rates With Increased Varenicline Dosage: A Propensity Score-Matched Analysis. *J Clin Psychopharmacol*. 2018;38(1):34-41. doi:10.1097/JCP.0000000000000829.
2. Cinciripini PM, Karam-Hage M, Kypriotakis G, et al. Association of a Comprehensive Smoking Cessation Program With Smoking Abstinence Among Patients With Cancer. *JAMA Netw Open*. 2019;2(9):e1912251. doi:10.1001/jamanetworkopen.2019.12251.
3. Hedeker D, Mermelstein RJ, Demirtas H. Analysis of binary outcomes with missing data: missing = smoking, last observation carried forward, and a little multiple imputation. *Addiction*. 2007;102(10):1564-1573. doi:10.1111/j.1360-0443.2007.01946.x.
4. Siddique J, Harel O, Crespi CM, Hedeker D. Binary variable multiple-model multiple imputation to address missing data mechanism uncertainty: application to a smoking cessation trial. *Statistics in medicine*. 2014;33(17):3013-3028. doi:10.1002/sim.6137.
5. White IR, Royston P. Imputing missing covariate values for the Cox model. *Statist. Med.* 2009;28(15):1982-1998. doi:10.1002/sim.3618.
6. CDC. Social Determinants of Health (SDOH) and PLACES Data | PLACES: Local Data for Better Health. Accessed June 18, 2024.

© 2024 Cinciripini PM et al. JAMA Oncology.
