# Supplementary material for: Survival Outcomes of an Early Intervention Smoking Cessation Treatment After a Cancer Diagnosis
Source: JAMA Oncol. 2024 Oct 31;10(12):1689–96. doi: 10.1001/jamaoncol.2024.4890 (PMC11528342; doi:10.1001/jamaoncol.2024.4890)
Supplement: Supplement 2. — Data Sharing Statement [file jamaoncol-e244890-s002.pdf]

Data Sharing Statement

Cinciripini. Early Intervention Smoking Cessation Treatment After a Cancer Diagnosis. *JAMA Oncol*. Published October 31, 2024. doi:10.1001/jamaoncol.2024.4890

Data
Data available: No

Additional Information
Explanation for why data not available: Data is not available for open sharing currently due to ongoing exploratory analyses alone and in combination with the PI's other data sets. Individual requests for anonymized data will be considered afterwards on a case-by-case basis.
